# Supplementary material for: Causal links of α-thalassemia indices and cardiometabolic traits and diabetes: MR study
Source: Life Sci Alliance. 2023 Oct 3;6(12):e202302204. doi: 10.26508/lsa.202302204 (PMC10547910; doi:10.26508/lsa.202302204)
Supplement: Supplementary file 1 [file LSA-2023-02204_TableS1.docx]

Supplementary Table 1. Chromosome 16p13.3 variants in 1493 Taiwan Biobank participants: Data derived from the whole genome sequence

| Chr | SNP | Position (GRCh37) | gene | Ref/Alt | Exon number | Exonic Function | mRNA sequence | AA Change. | MAF# | | | | |
| --- | --- | --- | --- | --- | --- | --- | --- | --- | --- | --- | --- | --- | --- |
|  |  |  |  |  |  |  |  |  | TWB | East Asian | Europe | American | African |
| 16 | rs191086839 | 186950 | *NPRL3* | T/C | -- | Intron Variant | -- | -- | 0.0177 | 0.0188 | <0.0001 | <0.0001 | <0.0001 |
| 16 | rs372755452 | 249621 | *LUC7L* | G>- | -- | Intron Variant | -- | -- | 0.0194 | 0.0218 | <0.0001 | <0.0001 | <0.0001 |
| 16 | rs375498857 | 427772 | *PGAP6* | C/A | 2 | Missense Variant | c.C198A | p.Arg66Ser | 0.0191 | 0.0208 | <0.0001 | <0.0001 | <0.0001 |

MAF (EU): data from 1000 Genome study, sample size: 1006 people.

Abbreviations: Chr, chromosome; Ref, reference allele; Alt, alter allele; HWE, Hardy-Weinberg equilibrium; MAF, minor allele frequency; AA, amino acid

#1000Genomes
