# Supplementary material for: Causal links of α-thalassemia indices and cardiometabolic traits and diabetes: MR study
Source: Life Sci Alliance. 2023 Oct 3;6(12):e202302204. doi: 10.26508/lsa.202302204 (PMC10547910; doi:10.26508/lsa.202302204)
Supplement: Supplementary file 2 [file LSA-2023-02204_TableS2.docx]

Supplementary Table 2. Stepwise linear regression analysis for microcytic traits and anemia, including genotypes

|  | MC_HC_E (1493) | | | | MC_HC_A (1493) | | | |
| --- | --- | --- | --- | --- | --- | --- | --- | --- |
|  | Beta | SE | *P* value | OR (95% CI) | Beta | SE | *P* value | OR (95% CI) |
| Age (years) | -0.0157 | 0.0124 | 0.2046 | 0.98 (0.96 - 1.01) | -0.0100 | 0.0127 | 0.4293 | 0.99 (0.97 - 1.01) |
| Sex (male *vs*. female) | 0.7546 | 0.3111 | 0.0153 | 2.13 (1.16 - 3.91) | 1.0527 | 0.3345 | 0.0017 | 2.87 (1.49 - 5.52) |
| Body mass index (kg/m^2^) | -0.0028 | 0.0379 | 0.9406 | 1.00 (0.93 - 1.07) | -0.0245 | 0.0403 | 0.5438 | 0.98 (0.90 – 1.06) |
| Current smoking (%) | -0.4418 | 0.6142 | 0.4720 | 0.64 (0.19 – 2.14) | 0.0261 | 0.5684 | 0.9634 | 1.03 (0.34 - 3.13) |
| *NPRL3* rs191086839 (TT *vs.*TC) | 3.7104 | 0.8814 | 2.56 × 10^-5^ | 40.87 (7.26 - 229.99) | 4.8884 | 1.2350 | 7.55 × 10^-5^ | 132.74 (11.80 - 1493.49) |
| *LUC7L* rs372755452 (GG *vs.* G-) | 3.0057 | 1.1471 | 0.0088 | 20.20 (2.13 - 191.32) | -1.8561 | 1.3556 | 0.1709 | 0.16 (0.01 - 2.23) |
| *PGAP6* rs375498857 (CC *vs.* CA) | 1.3135 | 1.1097 | 0.2366 | 3.74 (0.42 - 32.74) | -0.2421 | 0.9728 | 0.8034 | 0.78 (0.12 - 5.28) |

Abbreviations: OR: odds ratio, CI: confidence interval. Other abbreviations and participant recruitment as in Table 2 and Figure 1.
