# Supplementary material for: Causal links of α-thalassemia indices and cardiometabolic traits and diabetes: MR study
Source: Life Sci Alliance. 2023 Oct 3;6(12):e202302204. doi: 10.26508/lsa.202302204 (PMC10547910; doi:10.26508/lsa.202302204)
Supplement: Supplementary file 3 [file LSA-2023-02204_TableS3.docx]

Supplementary Table 3. The sensitivity, specificity, positive predictive values (PPV) and negative predictive values (NPV) of chromosome 16p13.3 variants for PCR confirmed α^0^ thalassemia deletion - -^SEA^ mutation

|  |  | α^0^ thalassemia deletion - -^SEA^ mutation | |  |  |  |
| --- | --- | --- | --- | --- | --- | --- |
| *NPRL3* rs191086839 |  | + | - | total |  |  |
| with minor allele | + | 51 | 1 | 52 | PPV | 98.08% |
|  | - | 9 | 1413 | 1422 | NPV | 99.37% |
|  | total | 60 | 1414 |  |  |  |
|  |  | sensitivity | specificity |  |  |  |
|  |  | 85.00% | 99.93% |  |  |  |
|  |  | α^0^ thalassemia deletion - -^SEA^ mutation | |  |  |  |
| *LUC7L* rs372755452 |  | + | - | total |  |  |
| with minor allele | + | 54 | 3 | 57 | PPV | 94.74% |
|  | - | 6 | 1411 | 1417 | NPV | 99.58% |
|  | total | 60 | 1414 |  |  |  |
|  |  | sensitivity | specificity |  |  |  |
|  |  | 90.00% | 99.79% |  |  |  |
|  |  | α^0^ thalassemia deletion - -^SEA^ mutation | |  |  |  |
| *PGAP6* rs375498857 |  | + | - | total |  |  |
| with minor allele | + | 51 | 5 | 56 | PPV | 91.07% |
|  | - | 9 | 1409 | 1418 | NPV | 99.37% |
|  | total | 60 | 1414 |  |  |  |
|  |  | sensitivity | specificity |  |  |  |
|  |  | 85.00% | 99.65% |  |  |  |
