# Supplementary material for: Causal links of α-thalassemia indices and cardiometabolic traits and diabetes: MR study
Source: Life Sci Alliance. 2023 Oct 3;6(12):e202302204. doi: 10.26508/lsa.202302204 (PMC10547910; doi:10.26508/lsa.202302204)
Supplement: Supplementary file 4 [file LSA-2023-02204_TableS4.docx]

Supplementary Table 4. Association of the *PGAP6* rs375498857 genotypes with metabolic and hematological phenotypes in TWB participants

| Clinical and laboratory parameters | N | Total | CC (112,202) | CA (3716) | AA (8) | beta | SE | *P* value* |
| --- | --- | --- | --- | --- | --- | --- | --- | --- |
| Anthropology |  |  |  |  |  |  |  |  |
| Age (years) | 115926 | 51 (40 - 59) | 51 (40 - 59) | 51 (41 - 59) | 48.5 (42.5 - 56.75) | 0.1350 | 0.1812 | 0.4561 |
| Waist circumference (cm) | 115863 | 83 (76 - 90) | 83 (76 - 90) | 82.1 (76 - 89) | 86.5 (71.625 - 89.625) | -0.0622 | 0.0842 | 0.4597 |
| Waist-hip ratio | 115859 | 0.87 (0.82 - 0.91) | 0.87 (0.82 - 0.91) | 0.87 (0.82 - 0.91) | 0.85 (0.79 - 0.89) | 0.0019 | 0.0009 | 0.0330 |
| Body mass index (kg/m^2^) | 115847 | 23.79 (21.56 - 26.35) | 23.80 (21.57 - 26.35) | 23.68 (21.46 - 26.11) | 25.87 (20.90 - 28.53) | -0.1756 | 0.0614 | 0.0043 |
| Blood Pressure |  |  |  |  |  |  |  |  |
| Systolic BP* (mmHg) | 101916 | 115.50 (105.33 - 127.00) | 115.5 (105.33 - 127.00) | 115.00 (105.50 - 127.00) | 115.50 (99.50 - 123.00) | -0.1836 | 0.2504 | 0.4633 |
| Diastolic BP* (mmHg) | 101916 | 71.00 (65.00 - 79.00) | 71.00 (65.00 - 79.00) | 71.00 (64.50 - 78.67) | 67.00 (62.00 - 85.00) | -0.2681 | 0.1638 | 0.1016 |
| Mean BP* (mmHg) | 101916 | 86.17 (78.83 - 94.56) | 86.17 (78.83 - 94.56) | 86.00 (78.5 - 94.545) | 88.17 (74.50 - 96.00) | -0.2400 | 0.1800 | 0.1825 |
| Lipid profiles |  |  |  |  |  |  |  |  |
| Total cholesterol# (mg/dL) | 107233 | 193.00 (171.00 - 217.00) | 193 (171 - 217) | 186 (165 - 211) | 202.5 (162.75 - 238.25) | -0.0164 | 0.0013 | 4.57 × 10^-36^ |
| HDL-cholesterol# (mg/dL) | 107233 | 53.00 (45.00 - 63.00) | 53 (45 - 63) | 52 (44 - 62) | 49.5 (39.25 - 58.5) | -0.0110 | 0.0016 | 1.92 × 10^-12^ |
| LDL-cholesterol# (mg/dL) | 107233 | 119.00 (99.00 - 141.00) | 119 (100 - 141) | 114 (95 - 135) | 133.5 (108.25 - 166.25) | -0.0198 | 0.0020 | 5.91 × 10^-24^ |
| Triglyceride# (mg/dL) | 107233 | 91.00 (64.00 - 133.00) | 91 (64 - 133) | 90 (63 - 131) | 86 (65.75 - 141.25) | -0.0042 | 0.0037 | 0.2487 |
| Glucose metabolism |  |  |  |  |  |  |  |  |
| Fasting plasma glucose** (mg/dL) | 109997 | 92.00 (87.00 - 97.00) | 92.00 (87.00 - 97.00) | 91.00 (87.00 - 96.00) | 89.00 (85.00 - 93.00) | -0.6022 | 0.2465 | 0.0146 |
| HbA1c** (%) | 109996 | 5.60 (5.40 - 5.80) | 5.60 (5.40 - 5.80) | 5.70 (5.50 - 6.00) | 5.70 (5.40 - 6.10) | 0.1306 | 0.0099 | 5.13 × 10^-40^ |
| Uric acid |  |  |  |  |  |  |  |  |
| Uric acid (mg/dL) | 111451 | 5.20 (4.40 - 6.20) | 5.20 (4.40 - 6.20) | 5.20 (4.30 - 6.10) | 5.60 (4.28 - 7.95) | -0.0164 | 0.0184 | 0.3739 |
| Renal function |  |  |  |  |  |  |  |  |
| Creatinine (mg/dL) | 115922 | 0.68 (0.58 - 0.84) | 0.68 (0.58 - 0.84) | 0.67 (0.57 - 0.82) | 0.68 (0.6225 - 0.96) | -0.0087 | 0.0046 | 0.0578 |
| eGFR (mL/min/1.73 m^2^) | 115913 | 100.07 (86.88 - 115.55) | 100.05 (86.84 - 115.54) | 101.29 (87.8225 - 117.28) | 91.19 (85.0875 - 100.83) | 1.3686 | 0.3586 | 1.36 × 10^-4^ |
| Albuminuria (mg/L) | 115729 | 8.7 (5.3 - 15.2) | 8.7 (5.3 - 15.2) | 8.6 (5.2 - 15) | 8.6 (4.3 - 17.925) | -7.1368 | 3.2153 | 0.0264 |
| Liver function |  |  |  |  |  |  |  |  |
| AST (U/L) | 115924 | 23 (20 - 27) | 23 (20 - 27) | 22 (19 - 27) | 20 (18 - 21.75) | -0.3868 | 0.2013 | 0.0546 |
| ALT (U/L) | 115764 | 19 (14 - 27) | 19 (14 - 27) | 18 (13 - 27) | 16.5 (12.75 - 20.75) | -0.5141 | 0.3287 | 0.1178 |
| gGT (U/L) | 115910 | 17 (12 - 26) | 17 (12 - 26) | 17 (12 - 26) | 14 (10 - 17.5) | -0.7368 | 0.5239 | 0.1597 |
| Serum albumin (g/dL) | 115924 | 4.5 (4.4 - 4.7) | 4.5 (4.4 - 4.7) | 4.5 (4.4 - 4.7) | 4.5 (4.225 - 4.675) | 0.0004 | 0.0037 | 0.9147 |
| Total bilirubin (mg/dL) | 115924 | 0.6 (0.5 - 0.8) | 0.6 (0.5 - 0.8) | 0.6 (0.5 - 0.775) | 0.55 (0.5 - 0.7) | -0.0344 | 0.0045 | 2.25 × 10^-14^ |
| Hematological parameters |  |  |  |  |  |  |  |  |
| Leukocyte count (10^3^/μL) | 115915 | 5.6 (4.7 - 6.7) | 5.6 (4.7 - 6.7) | 5.7 (4.8 - 6.8) | 6.5 (5.625 - 7.75) | 0.0311 | 0.4909 | 0.9495 |
| Hematocrit (%) | 115915 | 41.5 (38.9 - 44.5) | 41.6 (39 - 44.5) | 39.9 (37.2 - 42.8) | 40.7 (37.975 - 40.8) | -1.5997 | 0.0568 | 9.01 × 10^-174^ |
| Platelet count (10^3^/μL) | 115913 | 238 (202 - 277) | 238 (202 - 277) | 240 (204 - 284) | 228 (137.75 - 325) | 4.3584 | 0.9667 | 7.00 × 10^-6^ |
| Red blood cell count (10^6^/μL) | 115915 | 4.69 (4.4 - 5.05) | 4.67 (4.39 - 5.01) | 5.74 (5.34 - 6.18) | 5.45 (5.0075 - 6.2375) | 1.0176 | 0.0068 | < 10^-307^ |
| Hemoglobin (g/dL) | 115915 | 13.7 (12.8 - 14.8) | 13.8 (12.9 - 14.9) | 12.5 (11.7 - 13.6) | 13 (11.5 - 14.05) | -1.1874 | 0.0203 | < 10^-307^ |
| MCH (pg/RBC) | 115915 | 29.75 (28.54 - 30.73) | 29.81 (28.71 - 30.77) | 21.47 (20.79 - 22.32) | 21.27 (20.75 - 25.74) | -7.1672 | 0.0422 | < 10^-307^ |
| MCHC (g/dL) | 115915 | 33.33 (32.28 - 34.06) | 33.33 (32.39 - 34.09) | 31.48 (30.73 - 32.24) | 32.34 (30.91 - 32.75) | -1.6369 | 0.0248 | < 10^-307^ |
| MCV (fL) | 115915 | 88.96 (85.63 - 92.18) | 89.14 (86.03 - 92.31) | 68.45 (66.11 - 71.43) | 67.69 (64.86 - 81.50) | -18.2699 | 0.1148 | < 10^-307^ |
| Atherosclerotic risk factors |  |  |  |  |  |  |  |  |
| Diabetes mellitus (%) | 10965 | 9.50% | 9.40% | 11.20% | 12.50% | 0.2631 | 0.0558 | 2.00 × 10^-6^ |
| Hypertension (%) | 25832 | 22.30% | 22.40% | 19.90% | 12.50% | -0.1372 | 0.0458 | 0.0028 |
| Current smoking (%) | 22801 | 19.70% | 19.70% | 19.90% | 50.00% | 0.0556 | 0.0472 | 0.2394 |
| Gout (%) | 4472 | 3.90% | 3.90% | 3.50% | 0.00% | -0.0751 | 0.0942 | 0.4251 |
| Microalbuminuria (%) | 12969 | 11.20% | 11.20% | 10.70% | 12.50% | -0.0256 | 0.0544 | 0.6381 |
| Metabolic syndrome (%) | 29592 | 25.50% | 25.60% | 23.20% | 37.50% | -0.1000 | 0.0447 | 0.0253 |

Abbreviations and participant recruitment as in Table 1 and Figure 1. *Adjustment for age, sex, current smoking status, and BMI.
