# Supplementary material for: Causal links of α-thalassemia indices and cardiometabolic traits and diabetes: MR study
Source: Life Sci Alliance. 2023 Oct 3;6(12):e202302204. doi: 10.26508/lsa.202302204 (PMC10547910; doi:10.26508/lsa.202302204)
Supplement: Supplementary file 5 [file LSA-2023-02204_TableS5.docx]

**Supplementary Table 5.** Summary of coefficients used for Mendelian randomization analysis: MCV and MCH for cardiometabolic traits

1. *NPRL3* rs191086839 as instrumental variable

| T_A_ | T_B_ | G_A_ | T_A_-T_B_ | | | G_A_-T_A_ | | | G_A_-T_B_ | | | IV_A_-T_B_ | | | IV_A_-T_B_-adjT_A_ | | |
| --- | --- | --- | --- | --- | --- | --- | --- | --- | --- | --- | --- | --- | --- | --- | --- | --- | --- |
|  |  |  | Beta | SE | *P^a^* | Beta | SE | *P^a^* | Beta | SE | *P^a^* | Beta | SE | *P* | Beta | SE | *P*^b^ |
| MCH | Total cholesterol# (mg/dL) | *NPRL3* rs191086839 | 0.0028 | 0.0001 | 9.07 × 10^-275^ | -7.61191 | 0.04712 | < 10^-307^ | -0.01847 | 0.00142 | 1.16 × 10^-38^ | 0.00243 | 0.00019 | 1.16 × 10^-38a^ (1.62 × 10^-40c^) | -0.00054 | 0.00021 | 0.00907 |
| MCH | HDL-cholesterol# (mg/dL) | *NPRL3* rs191086839 | 0.0027 | 0.0001 | 6.33 × 10^-183^ | -7.61191 | 0.04712 | < 10^-307^ | -0.01369 | 0.00170 | 6.96 × 10^-16^ | 0.00180 | 0.00022 | 6.96 × 10^-16 a^ (3.62 × 10^-11c^) | -0.00126 | 0.00025 | 4.12 × 10^-7^ |
| MCH | LDL-cholesterol# (mg/dL) | *NPRL3* rs191086839 | 0.0027 | 0.0001 | 1.44 × 10^-117^ | -7.61191 | 0.04712 | < 10^-307^ | -0.02299 | 0.00212 | 2.71 × 10^-27^ | 0.00302 | 0.00028 | 2.71 × 10^-17a^ (8.52 × 10^-29c^) | 0.00032 | 0.00031 | 0.30603 |
| MCH | HbA1c** (%) | *NPRL3* rs191086839 | -0.0150 | 0.0006 | 4.88 × 10^-136^ | -7.62092 | 0.04620 | < 10^-307^ | 0.14389 | 0.01068 | 2.37 × 10^-41^ | -0.01888 | 0.00140 | 2.37 × 10^-41a^ (1.16 × 10^-45d^) | -0.00542 | 0.00157 | 0.00054 |
| MCH | Total bilirubin (mg/dL) | *NPRL3* rs191086839 | 0.0087 | 0.0003 | 7.47 × 10^-219^ | -7.61262 | 0.04499 | < 10^-307^ | -0.03900 | 0.00490 | 1.79 × 10^-15^ | 0.00512 | 0.00064 | 1.79 × 10^-15a^ (3.79 × 10^-14e^) | -0.00471 | 0.00072 | 5.44 × 10^-11^ |
| MCH | DM | *NPRL3* rs191086839 | -0.0461 | 0.0036 | 1.72 × 10^-37^ | -7.61262 | 0.04499 | < 10^-307^ | 0.25961 | 0.06063 | 1.90 × 10^-5^ | -0.03410 | 0.00796 | 0.00002^a^ (0.00236^d^) | 0.01607 | 0.00908 | 0.07666 |
| T_A_ | T_B_ | G_A_ | T_A_-T_B_ | | | G_A_-T_A_ | | | G_A_-T_B_ | | | IV_A_-T_B_ | | | IV_A_-T_B_-adjT_A_ | | |
|  |  |  | Beta | SE | *P^a^* | Beta | SE | *P^a^* | Beta | SE | *P^a^* | Beta | SE | *P* | Beta | SE | *P*^b^ |
| MCV | Total cholesterol# (mg/dL) | *NPRL3* rs191086839 | 0.0008 | 0.00003 | 2.34 × 10^-150^ | -19.48948 | 0.12792 | < 10^-307^ | -0.01847 | 0.00142 | 1.16 × 10^-38^ | 0.00095 | 0.00007 | 1.16 × 10^-38a^ (1.62 × 10^-40c^) | 0.00021 | 0.00008 | 0.00956 |
| MCV | HDL-cholesterol# (mg/dL) | *NPRL3* rs191086839 | 0.0009 | 0.00004 | 4.88 × 10^-127^ | -19.48948 | 0.12792 | < 10^-307^ | -0.01369 | 0.00170 | 6.96 × 10^-16^ | 0.00070 | 0.00009 | 6.96 × 10^-16 a^ (3.62 × 10^-11c^) | -0.00020 | 0.00010 | 0.03388 |
| MCV | LDL-cholesterol# (mg/dL) | *NPRL3* rs191086839 | 0.0008 | 0.00004 | 6.74 × 10^-77^ | -19.48948 | 0.12792 | < 10^-307^ | -0.02299 | 0.00212 | 2.71 × 10^-27^ | 0.00118 | 0.00011 | 2.71 × 10^-17a^ (8.52 × 10^-29c^) | 0.00043 | 0.00012 | 0.00034 |
| MCV | HbA1c** (%) | *NPRL3* rs191086839 | -0.0075 | 0.0002 | 3.90 × 10^-245^ | -19.49675 | 0.12570 | < 10^-307^ | 0.14389 | 0.01068 | 2.37 × 10^-41^ | -0.00738 | 0.00055 | 2.37 × 10^-41a^ (1.16 × 10^-45d^) | 0.00011 | 0.00060 | 0.85735 |
| MCV | Total bilirubin (mg/dL) | *NPRL3* rs191086839 | 0.0018 | 0.0001 | 1.50 × 10^-66^ | -19.46998 | 0.12258 | < 10^-307^ | -0.03900 | 0.00490 | 1.79 × 10^-15^ | 0.00200 | 0.00025 | 1.79 × 10^-15a^ (3.79 × 10^-14e^) | 0.00034 | 0.00028 | 0.22792 |
| MCV | DM | *NPRL3* rs191086839 | -0.0233 | 0.0013 | 3.28 × 10^-69^ | -19.46998 | 0.12258 | < 10^-307^ | 0.25961 | 0.06063 | 1.90 × 10^-5^ | -0.01333 | 0.00311 | 0.00002^a^ (0.00236^d^) | 0.01302 | 0.00348 | 0.00019 |

1. *LUC7L* rs372755452 as instrumental variable

| T_A_ | T_B_ | G_A_ | T_A_-T_B_ | | | G_A_-T_A_ | | | G_A_-T_B_ | | | IV_A_-T_B_ | | | IV_A_-T_B_-adjT_A_ | | |
| --- | --- | --- | --- | --- | --- | --- | --- | --- | --- | --- | --- | --- | --- | --- | --- | --- | --- |
|  |  |  | Beta | SE | *P^a^* | Beta | SE | *P^a^* | Beta | SE | *P^a^* | Beta | SE | *P* | Beta | SE | *P*^b^ |
| MCH | Total cholesterol# (mg/dL) | *LUC7L* rs372755452 | 0.0028 | 0.0001 | 9.07 × 10^-275^ | -7.52015 | 0.04366 | < 10^-307^ | -0.01734 | 0.00132 | 3.51 × 10^-39^ | 0.00231 | 0.00018 | 3.51 × 10^-39a^ (1.34 × 10^-40c^) | -0.00069 | 0.00020 | 0.00048 |
| MCH | HDL-cholesterol# (mg/dL) | *LUC7L* rs372755452 | 0.0027 | 0.0001 | 6.33 × 10^-183^ | -7.52015 | 0.04366 | < 10^-307^ | -0.01233 | 0.00158 | 6.25 × 10^-15^ | 0.00164 | 0.00021 | 6.25 × 10^-15a^ (2.33 × 10^-10c^) | -0.00147 | 0.00024 | 6.11E-10 |
| MCH | LDL-cholesterol# (mg/dL) | *LUC7L* rs372755452 | 0.0027 | 0.0001 | 1.44 × 10^-117^ | -7.52015 | 0.04366 | < 10^-307^ | -0.02156 | 0.00198 | 1.33 × 10^-27^ | 0.00287 | 0.00026 | 1.33 × 10^-27a^ (7.52 × 10^-29c^) | 0.00015 | 0.00030 | 0.62043 |
| MCH | HbA1c** (%) | *LUC7L* rs372755452 | -0.0150 | 0.0006 | 4.88 × 10^-136^ | -7.51768 | 0.04286 | < 10^-307^ | 0.14354 | 0.00996 | 4.95 × 10^-47^ | -0.01909 | 0.00133 | 4.95 × 10^-47a^ (5.41 × 10^-52d^) | -0.00554 | 0.00150 | 0.00022 |
| MCH | Total bilirubin (mg/dL) | *LUC7L* rs372755452 | 0.0087 | 0.0003 | 7.47 × 10^-219^ | -7.51255 | 0.04171 | < 10^-307^ | -0.03640 | 0.00457 | 1.66 × 10^-15^ | 0.00485 | 0.00061 | 1.66 × 10^-15a^ (7.34 × 10^-14e^) | -0.00505 | 0.00069 | 1.69E-10 |
| MCH | DM | *LUC7L* rs372755452 | -0.0461 | 0.0036 | 1.72 × 10^-37^ | -7.51255 | 0.04171 | < 10^-307^ | 0.25824 | 0.05649 | 5.00 × 10^-6^ | -0.03437 | 0.00752 | 5.00 × 10^-6a^ (0.00083^d^) | 0.01567 | 0.00871 | 0.07199 |
| T_A_ | T_B_ | G_A_ | T_A_-T_B_ | | | G_A_-T_A_ | | | G_A_-T_B_ | | | IV_A_-T_B_ | | | IV_A_-T_B_-adjT_A_ | | |
|  |  |  | Beta | SE | *P^a^* | Beta | SE | *P^a^* | Beta | SE | *P^a^* | Beta | SE | *P* | Beta | SE | *P*^b^ |
| MCV | Total cholesterol# (mg/dL) | *LUC7L* rs372755452 | 0.0008 | 0.00003 | 2.34 × 10^-150^ | -19.21820 | 0.11866 | < 10^-307^ | -0.01734 | 0.00132 | 3.51 × 10^-39^ | 0.0009 | 0.00007 | 3.51 × 10^-39a^ (1.34 × 10^-40c^) | 0.00016 | 0.00008 | 0.04386 |
| MCV | HDL-cholesterol# (mg/dL) | *LUC7L* rs372755452 | 0.0009 | 0.00004 | 4.88 × 10^-127^ | -19.21820 | 0.11866 | < 10^-307^ | -0.01233 | 0.00158 | 6.25 × 10^-15^ | 0.00064 | 0.00008 | 6.25 × 10^-15a^ (2.33 × 10^-10c^) | -0.00028 | 0.00009 | 0.00233 |
| MCV | LDL-cholesterol# (mg/dL) | *LUC7L* rs372755452 | 0.0008 | 0.00004 | 6.74 × 10^-77^ | -19.21820 | 0.11866 | < 10^-307^ | -0.02156 | 0.00198 | 1.33 × 10^-27^ | 0.00112 | 0.00010 | 1.33 × 10^-27a^ (7.52 × 10^-29c^) | 0.00037 | 0.00012 | 0.00140 |
| MCV | HbA1c** (%) | *LUC7L* rs372755452 | -0.0075 | 0.0002 | 3.90 × 10^-245^ | -19.19834 | 0.11673 | < 10^-307^ | 0.14354 | 0.00996 | 4.95 × 10^-47^ | -0.00748 | 0.00052 | 4.95 × 10^-47a^ (5.41 × 10^-52d^) | 0.00003 | 0.00058 | 0.95796 |
| MCV | Total bilirubin (mg/dL) | *LUC7L* rs372755452 | 0.0018 | 0.0001 | 1.50 × 10^-66^ | -19.17470 | 0.11376 | < 10^-307^ | -0.03640 | 0.00457 | 1.66 × 10^-15^ | 0.0019 | 0.00024 | 1.66 × 10^-15a^ (7.34 × 10^-14e^) | 0.00023 | 0.00027 | 0.39803 |
| MCV | DM | *LUC7L* rs372755452 | -0.0233 | 0.0013 | 3.28 × 10^-69^ | -19.17470 | 0.11376 | < 10^-307^ | 0.25824 | 0.05649 | 5.00 × 10^-6^ | -0.01347 | 0.00295 | 5.00 × 10^-6a^ (0.00083^d^) | 0.01298 | 0.00334 | 0.00010 |

T_A_ and T_B_: phenotypes A (MCV and MCH level) and B (Total cholesterol, HDL-C, LDL-C, HbA1c, total bilirubin, and DM); G_A_: genotypes determining T_A_; IV_A_: instrumental variables for G_A_.

*a*: Adjustment for age, sex, current smoking status, and BMI

*b:* Adjustment for MCV or MCH level.

*c:* Adjustment for age, sex, current smoking status, BMI, and other possible confounders, such as systolic blood pressure (BP), hemoglobin A1C (HbA1c), uric acid (UA), estimated glomerular filtration rate (eGFR), aspartate aminotransferase (AST), and total bilirubin.

*d*: Adjustment for age, sex, current smoking status, BMI, and other possible confounders, such as systolic BP, total cholesterol, UA, eGFR, AST and total bilirubin.

*e*: Adjustment for age, sex, current smoking status, BMI, and other possible confounders, such as systolic BP, total cholesterol, HbA1c, UA, eGFR and AST.
